# A Microfabricated Platform for Generating Physiologically-Relevant Hepatocyte Zonation

**DOI:** 10.1038/srep26868

**Published:** 2016-05-31

**Authors:** William J. McCarty, O. Berk Usta, Martin L. Yarmush

**Affiliations:** 1 Center for Engineering in Medicine, Department of Surgery, Massachusetts General Hospital, Harvard Medical School, and Shriners Hospitals for Children-Boston, Boston, MA, USA

## Abstract

*In vitro* liver models have been important tools for more than 40 years for academic research and preclinical toxicity screening by the pharmaceutical industry. Hepatocytes, the highly metabolic parenchymal cells of the liver, are efficient at different metabolic chemistries depending on their relative spatial location along the sinusoid from the portal triad to the central vein. Although replicating hepatocyte metabolic zonation is vitally important for physiologically-relevant *in vitro* liver tissue and organ models, it is most often completely overlooked. Here, we demonstrate the creation of spatially-controlled zonation across multiple hepatocyte metabolism levels through the application of precise concentration gradients of exogenous hormone (insulin and glucagon) and chemical (3-methylcholanthrene) induction agents in a microfluidic device. Observed gradients in glycogen storage via periodic acid-Schiff staining, urea production via carbamoyl phosphatase synthetase I staining, and cell viability after exposure to allyl alcohol and acetaminophen demonstrated the *in vitro* creation of hepatocyte carbohydrate, nitrogen, alcohol degradation, and drug conjugation metabolic zonation. This type of advanced control system will be crucial for studies evaluating drug metabolism and toxicology using *in vitro* constructs.

*In vitro* liver models are important tools that have been used in various forms for more than 40 years for basic science and translational academic research and preclinical toxicity screening by the pharmaceutical industry. Hepatocytes are highly metabolic parenchymal liver cells that perform the carbohydrate metabolism, nitrogen metabolism, alcohol degradation, and drug conjugation functions, among others, of the liver. Monolayer cultures of homogenously-treated hepatocytes have traditionally been used as a first-order approximation of liver tissue^1^,^2^. However, the metabolism of hepatocytes is not uniform across the liver.

Hepatocytes are efficient at different metabolic processes depending on their relative spatial location along the sinusoid. This zonation of distinct metabolisms occurs from the portal triad blood supply (periportal or zone 1) to the venous drain (pericentral, perivenous, or zone 3) with intermediate or zone 2 cells in between^3^,^4^,^5^,^6^. The importance of recapitulating this hepatocyte metabolic heterogeneity *in vitro* to create more physiological liver models has been acknowledged for at least 20 years^7^. Zone 1 hepatocytes are efficient at glucose release from glycogen stores and pyruvate during the post-absorptive phase^8^,^9^,^10^, urea formation from ammonia and amino acid breakdown^10^,^11^, cholesterol biosynthesis^12^, and phase II conjugation of xenobiotics into polar entities for excretion^13^,^14^,^15^,^16^. Zone 3 hepatocytes are efficient at glucose uptake and storage as glycogen^10^,^17^, glutamine formation from ammonia^10^,^11^,^18^, alcohol degradation^19^, and phase I drug conjugation of xenobiotic compounds via cytochrome P450 monooxygenases^14^,^20^,^21^,^22^,^23^,^24^. This change in metabolism occurs over the length of the sinusoid, which contains approximately 25 cells^2^.

Despite the distinct metabolic signatures of hepatocytes in different zones, *in vitro* systems modelling the liver, including both static and flow plate and microfluidic devices, typically ignore these large metabolic gradients. While several papers have reported success using cell consumption-based oxygen gradients^25^,^26^,^27^ to mimic selected zonated hepatocyte functions across a large number of cells *in vitro*, it is increasingly clear that replication of the physical environment within the liver, the parameters classically understood to induce zonation^8^,^28^, is not sufficient to achieve zonation across many metabolic functions^29^. The underlying spatial differences in hepatocyte genetic expression that are initially defined during development play a dominant role^30^,^31^, suggesting that the direct control of hepatocyte function using hormone or chemical stimulation rather than consumption-based oxygen gradients, may be necessary to create *in vitro* zonation of multiple metabolisms.

Here, we demonstrate the creation of spatially-controlled zonation across multiple hepatocyte metabolisms *in vitro*, including examples of carbohydrate, nitrogen, alcohol degradation, and drug conjugation metabolisms, through the controlled application of concentration gradients of exogenous hormone and chemical induction agents, independent of hepatocyte consumption. In addition to recapitulating the zonation of glucose storage and urea production, this *in vitro* model mimics the zonal toxicity responses of the liver to example environmental agents and pharmaceuticals.

## Results

### Validation of the concentration and flow pattern

**Figure 1:**
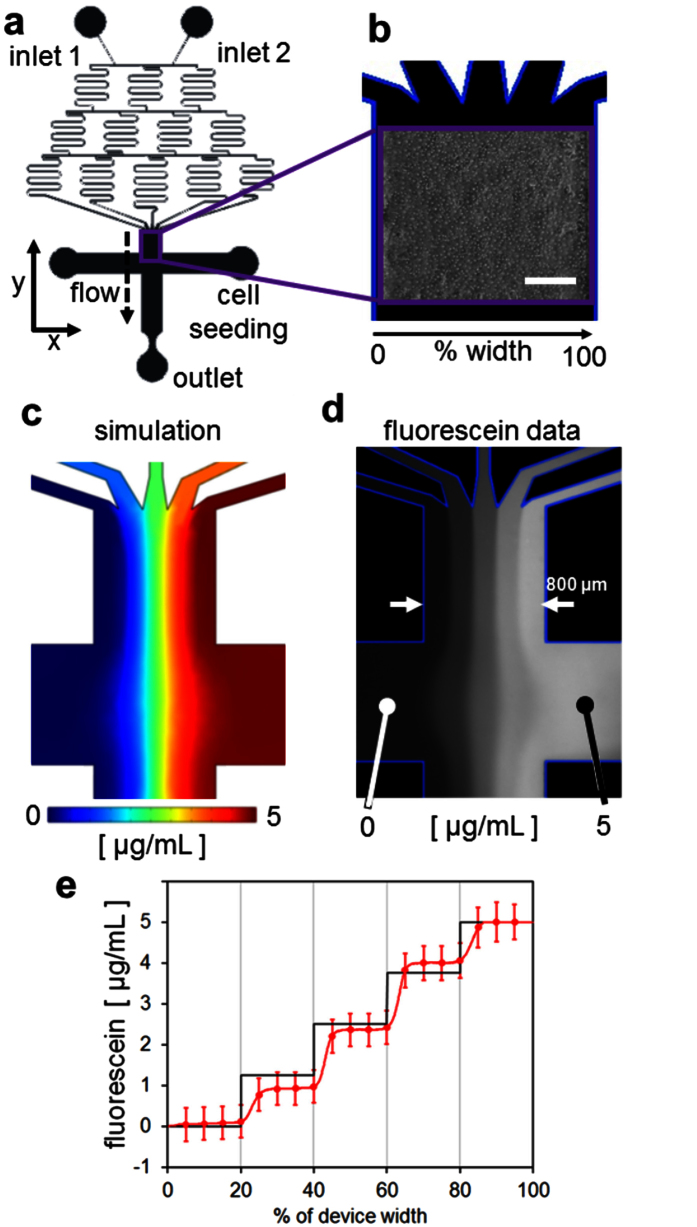
Device design, seeding, and the simulated and experimental concentration and flow patterns. (**a)** AutoCAD schematic of the device design showing the Christmas tree gradient connecting two inlets to the cell culture field. Left and right cell seeding ports were blocked after seeding so that flow occurs in the negative y-direction to the outlet. (**b)** Example phase image of a confluent field of hepatocytes seeded in the device with % width defined from left to right. (**c)** COMSOL simulation of and (**d)** experimental data for fluorescein (inlet 1: 0 μg/mL, 0.5 μL/min; inlet 2: 5 μg/mL, 0.5 μL/min). Bar: 200 μm. (**e)** Fluorescein concentration quantified across the device width based on n = 3 devices. The black line shows the target concentrations and the red the experimental data.

Microfluidic devices with a two inlet Christmas tree gradient were fabricated to allow the creation of a gradient of soluble factors based on the inlet concentrations across the main cell culture channel after seeding and attachment of primary rat hepatocytes (Fig. 1a,b). The concentration and flow patterns predicted using numerical simulation were very similar to those found experimentally. Both the numerical (Fig. 1c) and experimental (Fig. 1d) data showed five concentration channels generated by mixing inlets 1 and 2 that were maintained along the channel length. The concentration profile was quantified using image analysis, as described below, which confirmed that the channel concentrations measured for n = 3 devices were within the standard deviation of the predicted values (Fig. 1e). The predicted expansion and contraction of the flow lines at the channel intersection was also confirmed experimentally.

### Carbohydrate metabolism

**Figure 2:**
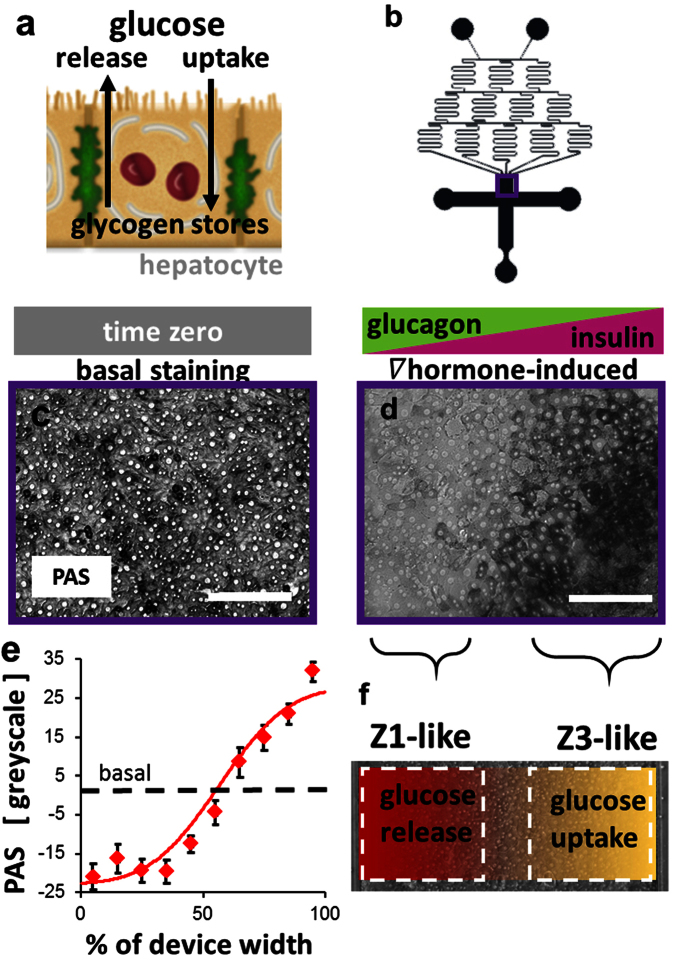
Zonation of carbohydrate metabolism *in vitro*. (**a)** During the absorptive phase, glucose is predominantly taken up and stored as glycogen by zone 3 hepatocytes, while during the post-absorptive phase, glucose is preferentially released by zone 1 hepatocytes. (**b)** Device design showing location of the images within the device. (**c)** PAS staining for glycogen in hepatocytes under basal conditions. (**d)** Glycogen in hepatocytes after 24 hr induction with a gradient of the insulin-to-glucagon ratio (inlet 1: 100 nM glucagon, 0 U/L insulin; inlet 2: 0 nM glucagon, 100 U/L insulin). (**e)** Quantification of glycogen staining intensity across the width of the device (n = 4 induced devices; repeated effect of location (% width): P < 0.001). (**f)** Diagram showing the zonation of carbohydrate metabolism induced across the field of hepatocytes. Bars: 200 μm.

During the absorptive phase, glucose is predominantly taken up and stored as glycogen by zone 3 hepatocytes, while during the post-absorptive phase, glucose is preferentially released by zone 1 hepatocytes^8^ (Fig. 2a). The hepatocytes were induced for 24 hr with basal media or media with a hormonal concentration gradient of the insulin-to-glucagon ratio. Channel-wide (Fig. 2b) representative images of the hepatocytes after PAS staining showed the basal (Fig. 2c) and hormone gradient-induced (Fig. 2d) glycogen storage. Quantification of the PAS staining across the width of the device relative to the basal staining intensity showed a gradient in glycogen storage (Fig. 2e, Supplemental Fig. 1a), demonstrating a gradient in carbohydrate metabolism (Fig. 2f).

### Nitrogen metabolism

**Figure 3:**
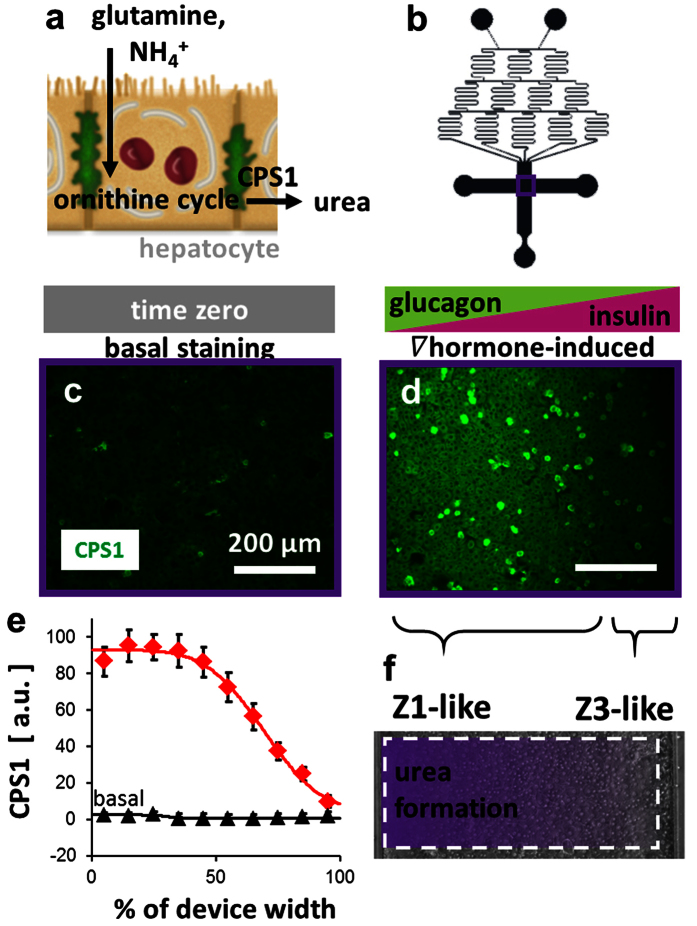
Zonation of nitrogen metabolism *in vitro*. (**a**) Glutamine and ammonia are predominantly taken up by zone 1 hepatocytes and converted to urea through the ornithine cycle, which depends on the activity of carbamoyl phosphate synthetase 1 (CPS1). (**b**) Device design showing location of the images within the device. (**c**) Staining for CPS1 in hepatocytes under basal conditions. (**d**) CPS1 in hepatocytes after 24 hr induction with a gradient of the insulin-to-glucagon ratio (inlet 1: 100 nM glucagon, 0 U/L insulin; inlet 2: 0 nM glucagon, 100 U/L insulin). (**e**) Quantification of CPS1 staining intensity across the width of the device (n = 7 basal, n = 6 induced devices; fixed effect of induction: P < 0.001; repeated effect of location (% width): P < 0.001). (**f**) Diagram showing the zonation of nitrogen metabolism induced across the field of hepatocytes. Bars: 200 μm.

Glutamine and ammonia are predominantly taken up by zone 1 hepatocytes and converted to urea through the ornithine cycle^29^, which depends on the activity of carbamoyl phosphate synthetase 1 (CPS1) (Fig. 3a). As above, the hepatocytes were induced for 24 hr with basal media or media with a hormonal concentration gradient of the insulin-to-glucagon ratio. Channel-wide (Fig. 3b) representative images of the hepatocytes after CPS1 staining showed the basal (Fig. 3c) and hormone gradient-induced (Fig. 3d) CPS1 intensities. Quantification of the CPS1 staining across the width of the device relative to the basal staining intensity (Fig. 3e) suggested a gradient in urea production (Supplemental Fig. 1b), demonstrating an *in vivo*-like gradient in nitrogen metabolism (Fig. 3f).

### Alcohol degradation

**Figure 4:**
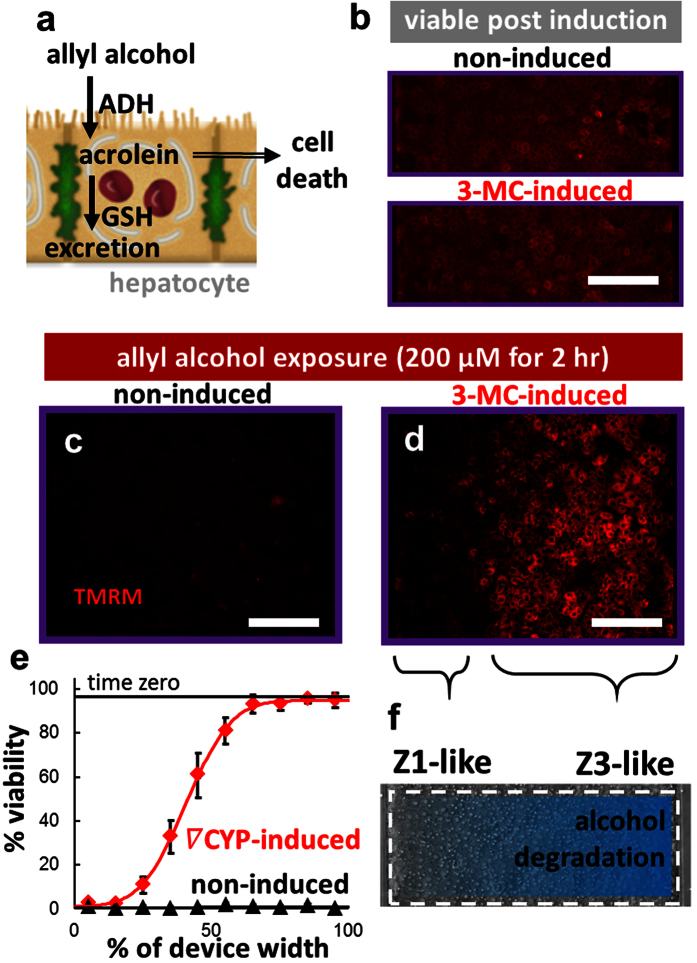
Zonation of alcohol degradation *in vitro*. (**a**) Allyl alcohol is oxidized by alcohol dehydrogenase (ADH) to the reactive aldehyde acrolein, which leads to preferential zone 1 cell death via lipid peroxidation if the protective phase II glutathione conjugation mechanism is overwhelmed. (**b**) After 24 hr induction with a gradient of 3-methylcholanthrene (3-MC) (inlet 1: 0 μM 3-MC, inlet 2: 2 μM 3-MC) or media (non-induced; inlet 1: media, inlet 2: media), the hepatocytes remained viable. After uniform exposure to an overdose of allyl alcohol (200 μM) for 2 hr, the (**c**) non-induced cells all died, while (**d**) induction led to a gradient in cell viability, indicating a gradient in protective alcohol degradation. (**e**) Quantification of viability across the width of the device (n = 6 non-induced, n = 6 induced devices; fixed effect of induction: P < 0.001; repeated effect of location (% width): P < 0.001). (**f**) Diagram showing the zonation of alcohol degradation induced across the field of hepatocytes. Bars: 200 μm.

In the liver, allyl alcohol is oxidized by alcohol dehydrogenase to the reactive aldehyde acrolein, which leads to preferential zone 1 cell death^32^ via lipid peroxidation if the protective phase II glutathione conjugation mechanism is overwhelmed^33^ (Fig. 4a). The hepatocytes seeded in each device were induced for 24 hr with basal media or media with a concentration gradient of 3-methylcholanthrene (3-MC). Here, the hepatocytes remained uniformly viable after gradient induction of glutathione conjugation with 3-MC^34^ (Fig. 4b). Representative images of the hepatocytes after uniform exposure to allyl alcohol showed complete cell death in the non-induced devices (Fig. 4c), but a gradient in viability after 3-MC induction (Fig. 4d). The quantified viability across the width of the device (Fig. 4e) suggested a gradient in glutathione conjugation activity, demonstrating an *in vivo*-like gradient in alcohol degradation (Fig. 4f).

### Drug conjugation metabolism

**Figure 5:**
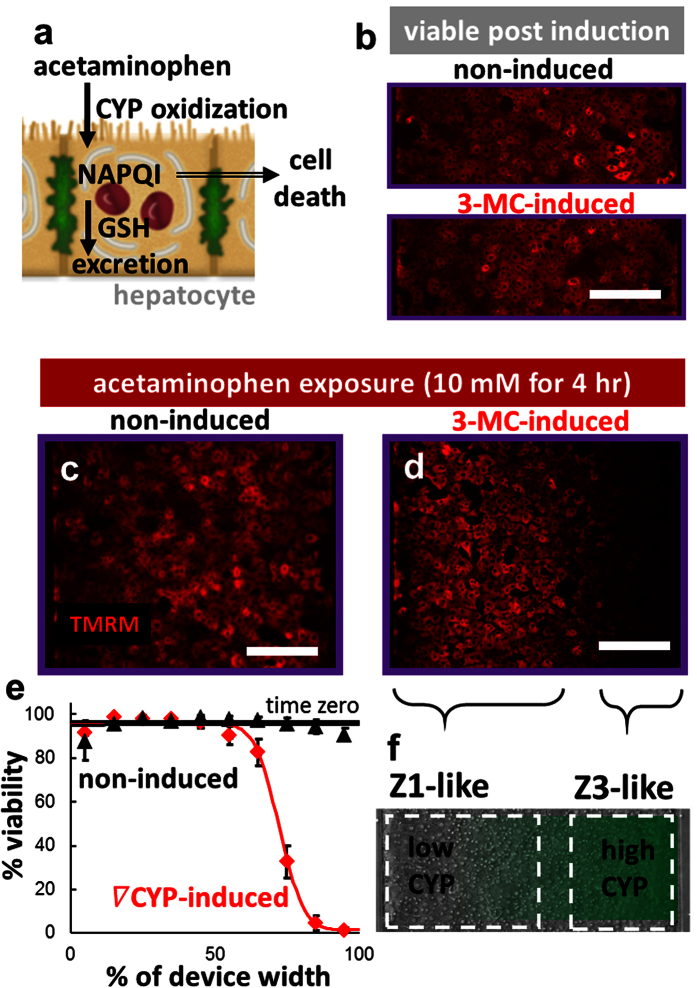
Zonation of drug conjugation metabolism *in vitro*. (**a**) Acetaminophen (APAP) is bioactivated by various CYP enzymes to the cytotoxic intermediate NAPQI, which depletes glutathione, leading to cell death after overdose. Only hepatocytes in zone 3 contain high CYP activity, making APAP a zone 3 toxin. (**b**) After 24 hr induction with a gradient of 3-methylcholanthrene (3-MC) (inlet 1: 0 μM 3-MC, inlet 2: 2 μM 3-MC) or media (non-induced; inlet 1: media, inlet 2: media), the hepatocytes remained viable. After uniform exposure to an overdose of APAP (10 mM) for 4 hr, the (**c**) non-induced cells all remained viable, while (**d**) induction led to a gradient in cell viability, indicating a gradient in CYP activity. (**e**) Quantification of viability across the width of the device (n = 6 non-induced, n = 6 induced devices; fixed effect of induction: P < 0.001; repeated effect of location (% width): P < 0.001). (**f**) Diagram showing the zonation of drug conjugation induced across the field of hepatocytes. Bars: 200 μm.

Acetaminophen (APAP) is bioactivated by various CYP enzymes in the liver to the cytotoxic intermediate NAPQI, which depletes glutathione, leading to cell death after overdose^35^. Only hepatocytes in zone 3 contain high CYP activity, making APAP a zone 3 toxin (Fig. 5a). The hepatocytes seeded in each device were induced for 24 hr with basal media or media with a concentration gradient of 3-methylcholanthrene. Here, the hepatocytes remained uniformly viable after gradient induction of CYP activity with 3-MC^34^ (Fig. 5b). Representative images of the hepatocytes after uniform exposure to APAP showed viable cells in the non-induced devices (Fig. 5c), but a gradient in viability after 3-MC induction (Fig. 5d). The quantified viability across the width of the device (Fig. 5e) suggested a gradient in CYP activity (Supplemental Fig. 1c), demonstrating an *in vivo*-like gradient in drug conjugation metabolism (Fig. 5f).

## Discussion

These results demonstrate the induction of gradients in a variety of hepatocyte metabolisms during *in vitro* culture within a microdevice. We modified hepatocyte metabolism through short-term (24 hr) concentration gradient induction with hormones (insulin and glucagon) or a chemical (3-MC) (Fig. 1). This induction produced a change from zone 1-like to zone 3-like hepatocyte metabolisms across approximately 25–40 cells, similar to the magnitude of change occurring along the sinusoid *in vivo*. The hormonal induction gradient produced hepatocytes with zonated glycogen storage (Fig. 2) and CPS1 content (Fig. 3), indicating zonation of carbohydrate and nitrogen metabolisms. The chemical induction gradient produced hepatocytes with zonated viability in response to glutathione-dependent zone 1 toxin allyl alcohol (Fig. 4) and CYP activity-dependent zone 3 toxin acetaminophen (Fig. 5), indicating zonation of alcohol degradation and drug conjugation metabolisms and providing example applications of the utility of the system.

There are several limitations to this study. First, we indirectly assessed changes in CYP and glutathione activities, urea production, and glucose release and uptake by hepatocytes in the devices by assessing the cell viability and CPS1 and glycogen staining intensities. However, pilot data in plates did show increases in CYP activity and urea production by hepatocytes cultured under similar conditions in plates (Supplemental Fig. 1). Unfortunately, those assays could not be easily translated to a continuous field of cells in a microdevice. Second, the hepatocyte metabolisms were assessed separately for the two inducers tested: carbohydrate and nitrogen metabolisms induced with hormones and alcohol degradation and drug conjugation metabolisms induced with 3-MC. Combining these inducers to create zonation across all metabolisms assessed would present a more physiological sinusoid. Third, the timeline of the experiments was limited by the loss of hepatocyte function over time that occurs when the cells are not cultured under a collagen gel^36^ or nanolayer assembly^37^. These experiments were performed within 48 hours of cell isolation, but some applications may require a longer time frame. Despite these limitations, the data presented here demonstrate creation of zonation in four categories of hepatocyte metabolism over a physiologically-relevant number of cells and length-scale.

The zonation of hepatocyte metabolism created here better mimics the metabolic functions of the liver sinusoid than static hepatocyte cultures by providing physically- and biologically-connected cells with spatially varying metabolic functions. These connections provide fields of cells with continuous changes in metabolic function over a physiologically-relevant number of cells (as demonstrated in Figs 2, 3, 4, 5) that can communicate with their neighbors, as occurs in the liver. Creating five independent cultures of cells that experience identical conditions as their neighbors may be able to replicate the average cellular metabolic response, but requires 5-fold more resources and precludes any physical or biological intercellular interaction between cells with varied metabolic responses, both of which occur between neighboring hepatocytes physiologically. Thus, the practical advantages of this approach over isolated culture systems include physical and biological coupling of cells with varied metabolic responses, spatial variation in the response to stimuli (Figs 4 and 5) – a hallmark of liver physiology, and increased throughput.

Beyond establishing the gradient in alcohol degradation (Fig. 4) and drug conjugation (Fig. 5) metabolisms, the data in Figs 4 and 5 are example applications that demonstrate ability of this system to distinguish likely modes of toxicity. For example, CYP-dependent toxicity occurs in Zone 3 hepatocytes because only those hepatocytes have enough CYP activity to create sufficiently high concentrations of toxic metabolites to cause cell death (Fig. 5). Similarly, direct toxins and those that require phase II-conjugation for safe elimination result in Zone 1 cell death (Fig. 4). Fundamental questions, such as the zonal region and likely mode of liver toxicity, can be answered in this culture system because of the spatial gradient in cell metabolism.

In contrast to the gradient of hepatocyte metabolisms established here, traditional static culture methods by design produce fields of cells uniform in function. For example, many methods of static hepatocyte culture include high levels of hormones, insulin in particular, and growth factors in the media, producing a uniform layer of zone 3-like cells. This leads to both under- and over-prediction of the hepatotoxicity of a drug depending on the mechanism. Zone 1 toxins, such as allyl alcohol (Fig. 4), may be under-reported if zone 3-like cells, containing increased glutathione conjugation and CYP activities, are used. On the other hand, over-prediction of LDH-leakage or other markers of hepatocyte toxicity may occur for zone 3 toxins, such as APAP (Fig. 5), because most hepatocytes in the liver lack the CYP activity necessary to create the toxic reactive intermediates. Similarly, the hepatocyte cultures with zonated carbohydrate and nitrogen metabolisms are more physiologically relevant culture systems than uniform hepatocyte monolayer cultures for basic science investigations because they provide spatially-controlled, heterogeneous metabolic function as is found in the liver.

In this device, the spatial gradient in metabolism is perpendicular to the flow direction, while in the liver the zonation of hepatocyte metabolism occurs along the flow direction. However, the underlying spatial differences in hepatocyte genetic expression initially defined during development play a dominant role^30^,^31^ in determining hepatocyte metabolism, with flow-direction consumption-based dynamic mediators, such as oxygen, playing a less important role. As a substitute for this spatial genetic control, we imposed precise chemical control of hepatocyte metabolism perpendicular to the flow direction, which allowed us to define cell metabolism independent of consumption. Further, to minimize any inadvertent effects of this cross-flow on the cells, the flow rate was chosen such that the shear stress induced had no effect on the cells^40^.

Hepatocyte cultures demonstrating metabolic zonation across a range of metabolisms may allow simultaneous testing of efficacy and toxicity for drugs targeting the liver. The liver plays central roles in both the oxidation of excess nitrogenous compounds, producing urea, and the regulation of blood glucose levels, providing glycogen stores that are a reservoir for glucose. Because of this, the liver is a common target for therapeutic interventions in both of these metabolic processes. For example, a culture system with zonated carbohydrate and drug conjugation metabolism can show the differential effects of a carbohydrate-modifying drug on hepatocytes at different locations along the sinusoid at the same time that the toxicity of the drug to zone 1 and zone 3 hepatocytes is defined.

Beyond the soluble hormones and chemical used here for induction, other categories of factors, such as genetic modulators, may produce effects over a wider range of hepatocyte expression. The genetic control of hepatocyte zonation is defined during development through the zone 1 localization of adenomatous polyposis coli, a negative regulator of Wnt/β-catenin signaling^30^. Wnt signaling imparts zone 3-like genetic expression to hepatocytes, while its inhibition imparts zone 1-like genetic expression^31^. Controlling Wnt/β-catenin signaling using the gradient induction microdevice demonstrated here may offer a simpler approach to spatially controlling the genetic profile of the hepatocytes.

In conclusion, we demonstrated the induction of spatially-controlled *in vitro* zonation in hepatocyte carbohydrate, nitrogen, alcohol degradation, and drug conjugation metabolisms. The zonation of hepatocyte metabolism is an often-overlooked, but vitally important aspect of physiologically-relevant *in vitro* liver tissue and organ models. As many disease states in the liver also exhibit zonation, the creation of zonated liver metabolisms will also be essential for the development of *in vitro* liver disease models.

## Methods

### Microfluidic device fabrication and preparation

Microfluidic devices were fabricated by replica molding PDMS from photolithographically defined SU-8 masters on silicon wafers. A 2-inlet, 5-channel Christmas tree gradient was used to allow creation of a gradient of the inlet concentrations across the main channel (Fig. 1a). PDMS was cast on the molds to a thickness of 2–3 mm and baked overnight at 70 °C. The main cell culture channel was 100 μm high, 800 μm wide, and 4 mm long. Inlet, cell seeding, and outlet ports were punched into the devices using 0.5 and 0.75 mm dermal punches. The devices were then plasma cleaned, bonded to microscope slides, baked (70 °C, 5 min), UV-sterilized (25 min), and coated with fibronectin (50 μg/mL, 37 °C, 45 min) in PBS before cell seeding.

### Hepatocyte isolation and culture

The protocols for the following experiments were approved by the Institutional Animal Care and Use Committee of Massachusetts General Hospital. The methods were carried out in accordance with the approved guidelines. Primary rat hepatocytes were freshly isolated from adult female Lewis rats (Charles River Laboratories, Wilmington, MA) weighing 180–200 g, by a modified procedure of Seglen^38^, as described previously^39^. Routinely, 200–300 million cells with 90–95% viability were isolated, as determined by trypan blue exclusion. Rat hepatocytes were seeded in Dulbecco’s modified eagle’s medium (Life Technologies, Carlsbad, CA, USA) supplemented with 10% fetal bovine serum (FBS, Sigma, St. Louis, MO, USA), 0.5 U/mL insulin, 7 ng/mL glucagon, 20 ng/mL epidermal growth factor, 7.5 μg/mL hydrocortisone, 200 U/mL penicillin, 200 μg/mL streptomycin, and 50 μg/mL gentamycin. The hepatocytes were seeded into devices (20 μL at 15 M cells/mL), allowed to attach for 3–4 hours, and then rinsed with fresh media. Finally, the cells were incubated overnight at 37 °C in humidified air with 10% CO_2_. Twenty-four hours after seeding, the cell media was replaced with basal, serum-free William’s E supplemented with 4 mM glutamine, 200 U/mL penicillin, and 200 μg/mL streptomycin (WEB media) that also contained various chemical and hormonal inducers as described below (Fig. 1b).

The total flow rate experienced by the cells during flow was 1.0 μL/min, with 0.5 μL/min moving through each inlet. This flow rate was chosen as sufficiently fast to prevent significant diffusive mixing of the channels, but also slow enough to limit the shear stress on the cells to less than 0.1 dynes/cm^2^. Shear stress values of 0.3 dynes/cm^2^ were previously shown to have no detrimental effects on cultured primary rat hepatocytes^40^.

### Zonation of hepatocyte carbohydrate and nitrogen metabolisms

#### Hormone Ratio Induction

Gradients in hepatocyte carbohydrate and nitrogen metabolisms were induced using an applied gradient in the ratio of insulin-to-glucagon. WEB media supplemented with 100 nM glucagon and 0 U/L insulin (inlet 1, 0.5 μL/min) and 0 nM glucagon and 100 U/L insulin (inlet 2, 0.5 μL/min) was pumped through devices placed inside an incubator for 24 hr.

#### Carbohydrate Metabolism

After induction with the hormone ratio gradient, periodic acid-Schiff (PAS) staining for glycogen following the manufacturer’s instructions (Sigma-Aldrich, St. Louis, MO) was used as a marker of glucose uptake and release by the hepatocytes in the devices. The average greyscale intensity of the PAS staining of each pixel across the width of each device was quantified on bright-field microscopy images using a custom image processing script (MATLAB, MathWorks, Natick, MA; ImageJ, NIH, Bethesda, MD). For each image, the script summed the greyscale intensities of each column of pixels to create a vector of total intensity at each pixel across the width of the device. The intensities were then normalized to the baseline controls and reported as a change in greyscale value.

#### Nitrogen Metabolism

After induction with the hormone ratio gradient, immunofluorescent staining for carbamoyl phosphate synthetase 1 (CPS1; 1:100, ab3682, abcam, Cambridge, MA) with an Alexa Fluor 488-conjugated secondary (1:200, ab150073, abcam) was used as a marker for the production of urea by the ornithine cycle. The total fluorescence intensity for each column of pixels across the width of each device was quantified using the custom image processing script above and normalized to the basal cell intensity as 0 a.u. and to the maximum total pixel intensity across the set of images as 100 a.u.

### Zonation of hepatocyte alcohol degradation and drug conjugation metabolisms

#### Chemical induction

Zonation of hepatocyte alcohol degradation and drug conjugation metabolisms was induced using an applied concentration gradient of 3-methylcholanthrene (3-MC). WEB media alone (inlet 1, 0.5 μL/min) and WEB supplemented with 2 μM 3-MC^37^ (inlet 2, 0.5 μL/min) was pumped through devices placed inside an incubator with the cells thus experiencing 1 μL/min flow for 24 hr. 3-MC induces the expression of CYP and glutathione drug conjugation enzymes in hepatocytes^34^.

#### Alcohol degradation

After induction with 3-MC, the flow was stopped and all the cells in each device were exposed to a toxic dose^41^ of the zone 1 toxin allyl alcohol (200 μM) for 2 hr at 37 °C. The average fluorescent signal at each pixel across the width of the device from tetramethylrhodamine methyl ester (TMRM; 500 nM in WEB, 20 min, 37 °C), which is sequestered by active mitochondria, was used as an indicator of cell viability. The images were automatically binarized in ImageJ, processed using the custom script as above, and normalized as a percent of the range between the positive and negative controls.

#### Drug conjugation metabolism

After induction with 3-MC, the flow was stopped and all the cells in each device were exposed to a toxic dose^42^ of the zone 3 toxin acetaminophen (10 mM in WEB) for 4 hr at 37 °C. The average fluorescent TMRM signal at each pixel across the width of the device was determined as above and used as an indicator of cell viability.

### Statistical Analysis

The data are presented as mean ± SEM for n = 4–7 devices or wells, derived from at least 3 different rat isolations. The effect of location (% device width) on PAS staining was assessed using 1-way repeated measures ANOVA. The fixed effect of induction (induced vs. non-induced) and repeated effect of location within the device (% device width) on cell viability or staining intensity were assessed using 2-way repeated measures ANOVAs. P-values less than 0.05 were considered statistically significant.

## Additional Information

**How to cite this article**: McCarty, W. J. *et al*. A Microfabricated Platform for Generating Physiologically-Relevant Hepatocyte Zonation. *Sci. Rep*. **6**, 26868; doi: 10.1038/srep26868 (2016).

## Supplementary Material

Supplementary Information
