# Supplementary material for: A Microfabricated Platform for Generating Physiologically-Relevant Hepatocyte Zonation
Source: Sci Rep. 2016 May 31;6:26868. doi: 10.1038/srep26868 (PMC4886516; doi:10.1038/srep26868)
Supplement: Supplementary Information [file srep26868-s1.pdf]

A Microfabricated Platform for Generating Physiologically-Relevant Hepatocyte Zonation

William J. McCarty[1], O. Berk Usta[1], and Martin L. Yarmush[1]

[1]Center for Engineering in Medicine, Department of Surgery, Massachusetts General Hospital, Harvard Medical School, and Shriners Hospitals for Children-Boston, Boston, MA, USA

Corresponding Author
Martin L. Yarmush
Center for Engineering in Medicine
51 Blossom St
Boston, MA 02114
Tel: (617) 726-3474
FAX: (617) 573-9471
Email: ireis@sbi.org

Supplementary Methods

Numerical simulation and experimental confirmation of the flow and concentration patterns

The concentration and flow patterns within the microdevice were simulated in COMSOL Multiphysics (COMSOL Inc., Burlington, MA, USA). The 3D device geometry was directly imported from the AutoCAD drawings used to print the photolithography mask. The simulated parameters included a flow rate of 0.5 µL/min into each inlet, a gauge pressure of 0 atm at the outlet, and an incoming concentration of 0 µg/mL fluorescein in the fluid at inlet 1 and 5 µg/mL at inlet 2. These same parameters were applied to an experimental device, which was imaged under a fluorescence microscope to visualize the concentration and flow patterns.

Carbohydrate metabolism pilot study

A proof-of-concept pilot study showed the effects of the insulin-to-glucagon ratio on glycogen storage in hepatocytes in static culture. Primary rat hepatocytes were cultured in 12-well tissue culture plates in WEB supplemented with various quantities of glucagon and insulin **(Supplemental Figure 1a)**. After 24 hours, the cells were fixed and stained with periodic acid-Schiff (PAS) stain for glycogen following the manufacturer's instructions (Sigma-Aldrich, St. Louis, MO). Digital images of each entire well were taken and the average greyscale intensity of the PAS staining of the cells for each well was quantified using a custom image processing script (MATLAB, MathWorks, Natick, MA; ImageJ, NIH, Bethesda, MD). Fields of representative images and the quantification of glycogen staining compared to baseline greyscale values are shown in supplementary **Supplemental Figure 1a**.

Nitrogen metabolism pilot study

A proof-of-concept pilot study showed the effects the glucagon on urea production by hepatocytes in static culture. Primary rat hepatocytes were cultured in 12-well tissue culture plates in WEB supplemented with various concentrations of glucagon **(Supplemental Fig. 1b)**. After 24 hours, the concentration of urea in the conditioned media from each well was assessed following the BUN Urea test kit from Stanbio Labs (Boerne, TX, USA).

Drug conjugation metabolism pilot study

A proof-of-concept pilot study showed the effects 3-methylcholanthrene (3-MC) on the activity of CYP1A1/2 in hepatocytes cultured in static conditions. Primary rat hepatocytes were cultured in 12-well tissue culture plates in WEB supplemented with various concentrations of 3-MC **(Supplemental Fig. 1c)** or vehicle control (0.1% DMSO). Native and induced CYP1A1/2 activities were determined by assessing the conversion of ethoxyresorufin to resorufin using fluorescence based on resorufin standards (EROD assay). The activities are reported as fold-induction by normalizing the induced values to the vehicle control values.

Statistical analysis

The effects of the insulin-to-glucagon ratio on PAS staining, glucagon concentration on urea production, and 3-MC induction on CYP activity were assessed using 1-way ANOVAs.

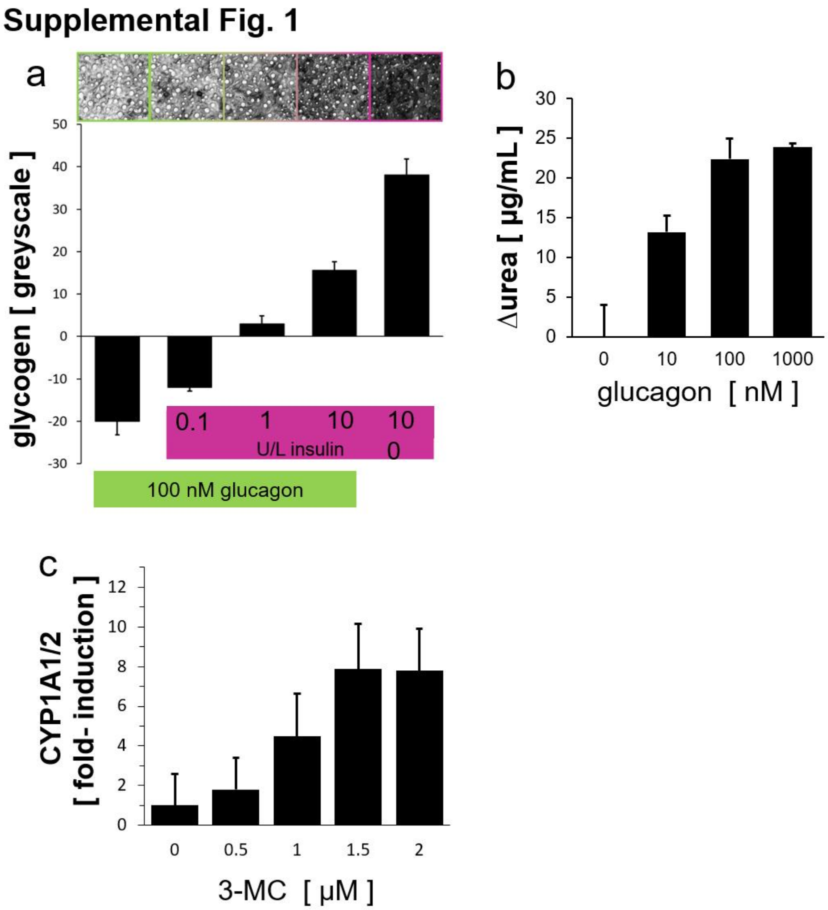

Supplemental Figure 1: Pilot studies showing the dose-response of hepatocyte metabolism in 12-well plates. a) PAS staining of hepatocytes incubated for 24 hr with insulin and glucagon indicating a significant effect (n=6 wells per group from 3 rat isolations; ANOVA: $P<0.001$) on glycogen storage. **b)** Change in the urea concentration in the conditioned media after 24 hr culture relative to the basal condition indicating a significant effect of glucagon (n=9 wells per group from 3 rat isolations; ANOVA: $P<0.01$). **c)** Fold-induction in CYP1A1/2 activity after 24 hr induction with 3-methylcholanthrene (3-MC) indicating a significant effect of 3-MC ($P<0.05$).
